# Remnant cholesterol is more positively related to diabetes, prediabetes, and insulin resistance than conventional lipid parameters and lipid ratios: A multicenter, large sample survey

**DOI:** 10.1111/1753-0407.13592

**Published:** 2024-08-13

**Authors:** Binqi Li, Yang Liu, Xin Zhou, Lulu Chen, Li Yan, Xulei Tang, Zhengnan Gao, Qin Wan, Zuojie Luo, Guijun Qin, Guang Ning, Weijun Gu, Yiming Mu

**Affiliations:** ^1^ School of Medicine Nankai University Tianjin China; ^2^ Department of Endocrinology First Medical Center of PLA General Hospital Beijing China; ^3^ Department of Endocrinology Eighth Medical Center of PLA General Hospital Beijing China; ^4^ Graduate School Chinese PLA General Hospital Beijing China; ^5^ Department of Medical Oncology Fifth Medical Center of Chinese PLA General Hospital Beijing China; ^6^ The Second Medical Center of Chinese PLA General Hospital Beijing China; ^7^ Wuhan Union Hospital Huazhong University of Science and Technology Wuhan China; ^8^ Department of Endocrinology Zhongshan University Sun Yat‐sen Memorial Hospital Guangzhou China; ^9^ Department of Endocrinology First Hospital of Lanzhou University Lanzhou China; ^10^ Department of Endocrinology Dalian Central Hospital Dalian China; ^11^ Department of Endocrinology Southwest Medical University Affiliated Hospital Luzhou China; ^12^ Department of endocrinology First Affiliated Hospital of Guangxi Medical University Nanning China; ^13^ Department of endocrinology First Affiliated Hospital of Zhengzhou University Zhengzhou China; ^14^ Department of Endocrinology, Ruijin Hospital Shanghai Jiao Tong University School of Medicine Shanghai China

**Keywords:** diabetes, insulin resistance, lipid ratios, prediabetes, remnant cholesterol

## Abstract

**Background:**

Not many large‐sample investigations are available that compare the potency of the relationship of remnant cholesterol (RC) and other lipid parameters with diabetes and prediabetes. The goals of our study are to discover the relationship between RC and prediabetes, diabetes, and insulin resistance (IR) and to investigate RC, high‐density lipoprotein cholesterol (HDL‐C), non‐HDL‐C, triglycerides (TG), low‐density lipoprotein cholesterol (LDL‐C), total cholesterol (TC), TC/HDL‐C, LDL‐C/HDL‐C, and TG/HDL‐C, which are the lipid parameters that are most positively related to diabetes, prediabetes, and IR.

**Methods:**

This research enrolled 36 684 subjects from China's eight provinces. We employed multiple logistic regression analysis for testing the relationship between lipid parameters and diabetes, prediabetes, and IR.

**Results:**

After adjusting for potential confounders, and comparing the results with other lipid parameters, the positive relationship between RC and diabetes (odds ratio [OR] 1.417, 95% confidence interval [CI]: 1.345–1.492), prediabetes (OR 1.555, 95% CI: 1.438–1.628), and IR (OR 1.488, 95% CI: 1.404–1.577) was highest. RC was still related to diabetes, prediabetes, and IR even when TG <2.3 mmol/L (diabetes: OR 1.256, 95% CI: 1.135–1.390; prediabetes: OR 1.503, 95% CI: 1.342–1.684; and IR: OR 1.278, 95% CI: 1.140–1.433), LDL‐C <2.6 mmol/L (diabetes: OR 1.306, 95% CI: 1.203–1.418; prediabetes: OR 1.597, 95% CI: 1.418–1.798; and IR: OR 1.552, 95% CI: 1.416–1.701), or HDL‐C ≥1 mmol/L (diabetes: OR 1.456, 95% CI: 1.366–1.550; prediabetes: OR 1.553, 95% CI: 1.421–1.697; and IR: OR 1.490, 95% CI: 1.389–1.598).

**Conclusion:**

RC is more positively related to diabetes, prediabetes, and IR than conventional lipids and lipid ratios in the general population, the relationships between RC and diabetes, prediabetes, and IR are stable, even if HDL‐C, LDL‐C, or TG are at appropriate levels.

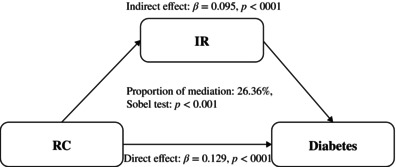

## Graphical abstract

Highlights

- This is the first investigation comparing remnant cholesterol (RC) and traditional lipids and lipid ratios with diabetes, prediabetes, and insulin resistance (IR) among a large, multicenter Chinese general population.
- RC is more positively related to diabetes, prediabetes, and IR than other conventional lipids and lipid ratios.
- The relationship between RC and diabetes, prediabetes, and IR are stable, even if conventional lipids are at appropriate levels.
- There is a significant indirect effect of IR between RC and diabetes and prediabetes with mediation.

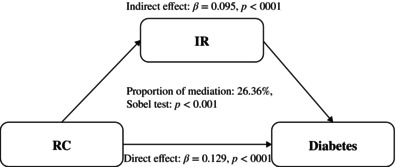

## 1 INTRODUCTION

Glycemic metabolism abnormalities, which include prediabetes and diabetes, appear to be increasingly prevalent. It was estimated that by 2045, the number of individuals with prediabetes^1^ would exceed 600 million, and those with diabetes^2^ would exceed 700 million worldwide. Prediabetes and diabetes are strongly connected to the risk of cardiovascular disease.^3^, ^4^, ^5^ In a recently conducted study^6^ involving 500 000 people in China, it was suggested that cardiovascular disease and hyperglycemia significantly raise the mortality risk. Hence, finding and managing the risk factors for prediabetes and diabetes is crucial.

Lipid metabolism and glucose metabolism are believed to be prevalent hazards for atherosclerosis, and they interact and are closely correlated.^7^ Unfortunately, the residual risk concerning atherosclerotic incidents among diabetic patients remains even if low‐density lipoprotein cholesterol (LDL‐C) concentrations are being maintained at prescribed limits.^8^ This suggests that conventional lipids, for instance LDL‐C, do not account for all hazards.^9^ Non‐traditional lipid indicators like remnant cholesterol (RC) deserve greater attention.

RC, triglyceride (TG)‐rich lipoprotein's (TGRL) metabolic residues, representing a group of cholesterol of different densities, volumes, and protein contents,^10^ specifically denotes metabolic residues of very low‐density lipoprotein (VLDL), intermediate‐density lipoprotein (IDL), and chylomicron during not‐empty‐stomach, as well as metabolic residues of VLDL and IDL during empty‐stomach.^10^ RC is now widely acknowledged as a key mediator of severe cardiovascular incidents.^11^, ^12^, ^13^ In 2021, the European Atherosclerosis Society reported that reducing TGRL and its residues (i.e., RC) is a strategic approach to prevent arteriosclerosis.^14^ However, there are fewer large‐sample investigations comparing the strength of the correlation of RC and other lipid parameters with diabetes and prediabetes, and in addition, there is currently an absence of studies on the relationship between RC and insulin resistance (IR) in the Chinese general population. The current study's goals are to find the relation between RC and prediabetes, diabetes, and IR, which is the prime reason for hyperglycemia, among a large‐sample, multicenter, Chinese general population as well as to compare the link between RC, conventional lipids, and lipid ratios and diabetes, prediabetes, and IR.

## 2 METHODS

### 2.1 Study design and subjects

Data for the current survey were obtained from eight centers (Lanzhou, Dalian, Zhengzhou, Nanning, Luzhou, Guangzhou, Wuhan, and Shanghai) of the^15^ Risk Evaluation of cAncers in Chinese diabeTic Individuals (REACTION) study. From March to December 2012, 53 639 Chinese people of ages 40 years and above participated in the study. After further exclusion of those with serious illnesses, use of lipid‐lowering medications or insulin within the past 30 days, missing significant data, and calculated RC <0, a total of 36 684 study participants were included. The REACTION study project was supported by Shanghai Jiaotong University's Medical Ethics Committee (No. 2011‐14). The project was executed based on the revised (1983) principles of the Declaration of Helsinki. Each participant provided informed consent in writing during every research visit.

### 2.2 Data acquisition

Trained researchers handed out standardized questionnaires and conducted direct interviews for gathering information about demographics, medical history, family history, and medication history. After participants took off their coats and shoes, height and weight were measured and noted. After a 5‐min break, systolic blood pressure (SBP) and diastolic blood pressure (DBP) were measured thrice at 5‐min intervals. Average value of these three measurements was taken to conduct the data analysis.

Participants abstained from eating for at least 8 h. Glycosylated hemoglobin (HbA1c) was detected through high‐performance liquid chromatography method. Total cholesterol (TC), LDL‐C, high‐density lipoprotein cholesterol (HDL‐C), TG, alanine transferase (ALT), aspartate transferase (AST), glutamine transferase (GGT), and creatinine were measured by autoanalyzer. After extracting fasting blood glucose (FBG), subjects not having diabetes underwent a 75 g oral glucose tolerance, subjects having diabetes underwent a 100 g steamed bread meal. Two‐hour postprandial BG (2‐h PBG) was obtained 2 h after the first intake of 75 g of glucose or 100 g of steamed bread. FBG and 2‐h PBG blood samples were collected in sodium fluoride tubes and measured using the hexokinase method.

### 2.3 Calculations and definitions

(1) RC = TC − HDL‐C–LDL‐C.^13^ (2) Non‐HDL‐C = TC−HDL‐C.^16^ (3) TG/HDL‐C, LDL‐C/HDL‐C, and TC/HDL‐C were captured by the corresponding lipid division.^17^ (4) Diabetes was defined under American Diabetes Association's (ADA)^18^ criteria: FBG ≥7.0 mmol/L or 2‐h PBG ≥11.1 mmol/L or HbA1c ≥6.5% or self‐reported diagnosis of diabetes. (5) Prediabetes was defined under ADA's^18^ standard: FBG <7.0 mmol/L and 2‐h PBG <11.1 mmol/L and HbA1c < 6.5% and (FBG ≥5.6 mmol/L or 2‐h PBG ≥7.8 mmo/L or HbA1c ≥ 5.7%). (6) Normal BG was defined as FBG <5.6 mmol/L and 2‐h PBG <7.8 mmol/L and HbA1c < 5.7%. (7) Homeostatic model of IR (HOMA‐IR)^19^ = FBG (mmol/L) × fasting insulin (mU/L)/22.5. IR was defined as HOMA‐IR values ≥75th percentile. (8) Estimated glomerular filtration rate (eGFR)^20^ (mL/min per 1.73 m^2^) = 175 × (SCr in mg/dL) − 1.154 × age − 0.203 × (0.742 for women) × (1.212 if African American). (9) Body mass index (BMI) (kg/m^2^) = weight (kg)/height^2^ (m).

### 2.4 Statistical analysis

We used SPSS 24.0 (IBM, Chicago, Illinois) for performing statistical analysis. Categorical variables were shown in numerical form (proportion). Continuous variables were shown in medians (interquartile range). Kruskal–Wallis rank sum test was used for detecting differences between continuous variables. Chi‐square test was used for testing the differences between categorical variables. Additionally, we used Spearman's test to initially explore correlations between continuous variables and used partial correlation analyses to explore correlations between continuous variables by grouping them according to gender, using age as a control variable.

Relationships of each lipid parameter with diabetes, prediabetes, and IR were investigated utilizing multiple logistic regression. The confounding factors were variables that showed differences across diabetic and nondiabetic groups, prediabetic and normal BG groups, or IR and non‐IR groups. Confounders' collinearity was detected before logistic regression.

The associations between RC and diabetes, prediabetes, and IR were investigated among gender, BMI, age, and blood pressure subgroups. Furthermore, depending on the suggested TG, HDL‐C, and LDL‐C's appropriate levels from guidelines for management of Chinese adults' dyslipidemia,^21^ the associations of RC and diabetes, prediabetes, and IR were also explored when LDL‐C <2.6 mmol/L, HDL‐C ≥1.0 mmol/L, and TG <2.3 mmol/L separately. Finally, given that IR was proven to be one of the major causes of hyperglycemia, we looked into whether IR had a mediating effect among the relationship of RC and diabetes and prediabetes. Effects of these mediators were evaluated using the bootstrap test.^22^ In addition, using the Yangtze River as the dividing line, the area south of the Yangtze River is categorized as southern China (Nanning, Luzhou, Guangzhou, Wuhan and Shanghai belong to southern China in this study), and the area north of the Yangtze River is categorized as northern China (Lanzhou, Dalian, Zhengzhou belong to northern China in this study). Southern and northern China have differences in economy, culture, food habits, and other factors. Previous multicenter studies^23^ have examined the differences in diabetes complications between north and south China. Therefore, we additionally performed a heterogeneity test to explore the association between RC and abnormal glucose metabolism in southern and northern China.

All statistical tests were two‐sided. A *p* < 0.05 was regarded as statistical significance.

## 3 RESULTS

### 3.1 Features of the subjects

The current survey contained 36,684 participants. Among the total population (Table 1), compared with subjects not having diabetes, those having diabetes were more advanced in age, more males, had higher RC, TG, TC, LDL‐C, TG/HDL‐C, LDL‐C/HDL‐C, TC/HDL‐C, non‐HDL‐C, ALT, AST, GGT, BMI, SBP, DBP, FBG, 2‐h PBG and HbA1c levels, and lower HDL‐C and eGFR, smoked cigarette more frequently, more had cardiovascular disease, and more had a family history of diabetes.

**TABLE 1:** Characteristics of the subjects by diabetes or prediabetes.

Variable	Total	All subjects		*p* value	No diabetes subjects		
		Diabetes	No diabetes		Prediabetes	Normal BG	*p* value
*N*	36 684	9416	27 268		20 442	6826	
Age, years	57 (52, 64)	60 (55, 68)	56 (51, 62)	<0.001	57 (52, 63)	54 (48, 59)	<0.001
Sex (%)				<0.001			<0.001
Male	11 321 (30.9)	3504 (37.2)	7817 (28.7)		5979 (29.2)	1838 (26.9)	
Female	25 363 (69.1)	5912 (62.8)	19 451 (71.3)		14 463 (70.8)	4988 (73.1)	
FBG, mmol/L	5.53 (5.11, 6.17)	7.01 (6.04, 8.40)	5.35 (5.01, 5.72)	<0.001	5.50 (5.14, 5.86)	5.06 (4.80, 5.30)	<0.001
2‐h PBG, mmol/L	7.35 (6.00, 9.63)	12.43 (10.12, 15.47)	6.70 (5.67, 7.95)	<0.001	7.11 (5.99, 8.39)	5.90 (5.10, 6.62)	<0.001
HbA1c, %	5.90 (5.60, 6.20)	6.70 (6.30, 7.50)	5.80 (5.50, 6.00)	<0.001	5.90 (5.70, 6.10)	5.40 (5.20, 5.50)	<0.001
HOMA‐IR	1.87 (1.29, 2.73)	2.73 (1.85, 4.11)	1.68 (1.19, 2.35)	<0.001	1.80 (1.27, 2.51)	1.39 (1.03, 1.87)	<0.001
RC, mmol/L	0.69 (0.49, 0.95)	0.55 (0.77, 1.08)	0.47 (0.66, 0.91)	<0.001	0.68 (0.49, 0.94)	0.60 (0.43, 0.82)	<0.001
LDL‐C, mmol/L	2.93 (2.36, 3.55)	2.96 (2.36, 3.58)	2.93 (2.36, 3.53)	0.013	2.97 (2.39, 3.58)	2.79 (2.28, 3.37)	<0.001
HDL‐C, mmol/L	1.29 (1.09, 1.52)	1.21 (1.03, 1.42)	1.32 (1.11, 1.55)	<0.001	1.31 (1.10, 1.53)	1.36 (1.15, 1.60)	<0.001
TC, mmol/L	5.05 (4.32, 5.80)	5.10 (4.33, 5.89)	5.04 (4.32, 5.76)	<0.001	5.10 (4.37, 5.83)	4.86 (4.19, 5.57)	<0.001
TG, mmol/L	1.37 (0.98, 1.97)	1.65 (1.15, 2.36)	1.29 (0.93, 1.83)	<0.001	1.35 (0.98, 1.91)	1.11 (0.83, 1.56)	<0.001
Non‐HDL‐C, mmol/L	3.70 (3.06, 4.39)	3.82 (3.14, 4.55)	3.66 (3.03, 4.34)	<0.001	3.73 (3.09, 4.42)	3.45 (2.86, 4.10)	<0.001
TG/HDL‐C	1.07 (0.69, 1.69)	1.38 (0.89, 2.12)	0.98 (0.65, 1.53)	<0.001	1.04 (0.69, 1.62)	0.83 (0.56, 1.27)	<0.001
TC/HDL‐C	3.89 (3.29, 4.56)	4.18 (3.57, 4.85)	3.79 (3.21, 4.45)	<0.001	3.88 (3.29, 4.54)	3.55 (3.03, 4.15)	<0.001
LDL‐C/HDL‐C	2.29 (1.83, 2.78)	2.44 (1.98, 2.94)	2.23 (1.78, 2.72)	<0.001	2.29 (1.84, 2.78)	2.08 (1.65, 2.53)	<0.001
BMI, kg/m^2^	24.27 (22.13, 26.57)	25.20 (23.12, 27.53)	21.84 (23.92, 26.21)	<0.001	24.18 (22.07, 26.45)	23.22 (21.26, 25.34)	<0.001
ALT, U/L	15 (11, 21)	16 (12, 24)	14 (10, 20)	<0.001	14 (11, 20)	14 (10, 19)	<0.001
AST, U/L	20 (17, 25)	21 (17, 26)	20 (17, 24)	<0.001	20 (17, 25)	20 (17, 24)	<0.001
GGT, U/L	21 (15, 32)	25 (17, 39)	19 (14, 29)	<0.001	20 (15, 30)	17 (13, 26)	<0.001
SBP, mmHg	129 (117, 144)	136 (123, 150)	127 (115, 141)	<0.001	129 (117, 143)	122 (112, 136)	<0.001
DBP, mmHg	77 (70, 84)	78 (71, 85)	76 (70, 83)	<0.001	77 (70, 84)	75 (68, 82)	<0.001
Creatinine	65.50 (9.40, 73.00)	67.30 (60.60, 76.40)	64.90 (59.10, 72.30)	<0.001	65.10 (59.20, 72.70)	64.10 (58.60, 71.30)	<0.001
eGFR	89.78 (80.09, 100.68)	87.80 (77.27, 99.73)	90.43 (81.09, 94.00)	<0.001	89.85 (80.45, 100.43)	91.91 (82.95, 102.16)	<0.001
Drinking (%)				0.664			0.119
Never	27 424 (74.8)	7141 (75.8)	20 283 (74.4)		15 216 (74.4)	5067 (74.2)	
Occasional	6791 (18.5)	1561 (16.6)	5230 (19.2)		3881 (19.0)	1349 (19.8)	
Frequently	2467 (6.7)	714 (7.6)	1755 (6.4)		1345 (6.6)	410 (6.0)	
Smoking (%)				<0.001			0.832
Never	31 477 (85.8)	7957 (84.5)	23 520 (86.3)		17 627 (86.2)	5893 (86.3)	
Occasional	864 (2.4)	247 (2.6)	617 (2.3)		469 (2.3)	148 (2.2)	
Frequently	4343 (11.8)	1212 (12.9)	3131 (11.5)		2346 (11.5)	785 (11.5)	
Cardiovascular disease (%)				<0.001			<0.001
Yes	1723 (4.7)	727 (7.7)	996 (3.7)		829 (4.1)	167 (2.4)	
No	34 961 (95.3)	8689 (92.3)	26 272 (96.3)		19 613 (95.9)	6659 (97.6)	
Family history of diabetes (%)				<0.001			<0.001
Yes	6428 (17.5)	2225 (23.6)	4203 (15.4)		3257 (15.9)	946 (13.9)	
No	30 256 (82.5)	7191 (76.4)	23 065 (84.6)		17 185 (84.1)	5880 (86.1)	

*Note*: Values were expressed as medians (quartile interval) or *n* (%).

Abbreviations: 2‐h PBG, 2‐h postprandial blood glucose; ALT, alanine transferase; AST, aspartate transferase; BMI, body mass index; DBP, diastolic blood pressure; eGFR, estimated glomerular filtration rate; FBG, fasting blood glucose; GGT, gamma‐glutamyl transferase; HbA1c, glycated hemoglobin; HDL‐C, high‐density lipoprotein cholesterol, HOMA‐IR, homeostasis model assessment of insulin resistance; LDL‐C, low‐density lipoprotein cholesterol; Non‐HDL‐C non‐high‐density lipoprotein‐cholesterol, RC, remnant cholesterol; SBP, systolic blood pressure; TC, total cholesterol; TG, triglyceride.

Among the nondiabetic population (Table 1), in comparison with subjects free from prediabetes, prediabetic subjects were more aged, more males, had higher RC, LDL‐C, TG, TC, TG/HDL‐C, LDL‐C/HDL‐C, TC/HDL‐C, non‐HDL‐C, higher BMI, ALT, AST, GGT, SBP, DBP, FBG, 2‐h PBG, and HbA1c levels, and lower HDL‐C and eGFR, more people had cardiovascular disease, and more had family history of diabetes.

Among the total population (Table 2), and comparing with subjects not having IR, subjects having IR were older, more males, had higher RC, FBG, 2‐h PBG, HbA1c, LDL‐C, TG, TC, TG/HDL‐C, LDL‐C/HDL‐C, TC/HDL‐C, non‐HDL‐C, BMI, ALT, AST, GGT, SBP, and DBP levels, and lower HDL‐C and eGFR, fewer frequent smokers, more people had cardiovascular diseases, and more had family history of diabetes.

**TABLE 2:** Characteristics of subjects with HOMA‐IR ≥75th percentile and HOMA‐IR <75th percentile.

Variable	All subjects		*p* value
	HOMA‐IR ≥75th percentile	HOMA‐IR <75th percentile	
*N*	9174	27 510	
Age, years	58.56 (53.06, 65.25)	56.88 (51.73, 62.94)	<0.001
Sex, %			<0.001
Male	2795 (30.5)	8526 (31.0)	
Female	6383 (69.5)	18 980 (69.0)	
FBG, mmol/L	6.30 (5.67, 7.72)	5.39 (5.01, 5.82)	<0.001
2‐h PBG, mmol/L	9.57 (7.37, 13.59)	6.90 (5.74, 8.63)	<0.001
HbA1c, %	6.20 (5.80, 7.00)	5.80 (5.50, 6.10)	<0.001
HOMA‐IR	3.66 (3.10, 4.72)	1.56 (1.15, 2.04)	<0.001
RC, mmol/L	0.81 (0.57, 1.13)	0.65 (0.47, 0.89)	<0.001
LDL‐C, mmol/L	3.02 (2.42, 3.64)	2.91 (2.34, 3.51)	0.013
HDL‐C, mmol/L	1.19 (1.02, 1.38)	1.33 (1.12, 1.56)	<0.001
TC, mmol/L	5.19 (4.43, 5.92)	5.01 (4.29, 5.75)	<0.001
TG, mmol/L	1.80 (1.29, 2.57)	1.25 (0.91, 1.77)	<0.001
Non‐HDL‐C, mmol/L	3.94 (3.29, 4.61)	3.63 (3.00, 4.30)	<0.001
TG/HDL‐C	1.52 (1.02, 2.30)	0.95 (0.63, 1.47)	<0.001
TC/HDL‐C	4.32 (3.73, 4.96)	3.75 (3.18, 4.40)	<0.001
LDL‐C/HDL‐C	2.52 (2.08, 3.01)	2.21 (1.76, 2.70)	<0.001
BMI, kg/m^2^	26.45 (24.46, 28.67)	23.57 (21.62, 25.71)	<0.001
ALT, U/L	18 (13, 27)	14 (10, 19)	<0.001
AST, U/L	21 (17, 27)	20 (17, 24)	<0.001
GGT, U/L	27 (19, 42)	19 (14, 28)	<0.001
SBP, mmHg	137 (124, 151)	127 (1157141)	<0.001
DBP, mmHg	80 (73, 87)	76 (69, 83)	<0.001
Creatinine	66.7 (60.5, 75.0)	65.1 (59.1, 72.6)	<0.001
eGFR	87.54 (77.59, 98.40)	90.54 (81.02, 101.31)	<0.001
Drinking, %			0.664
Never	7100 (77.4)	20 324 (73.9)	
Occasional	1477 (16.1)	5314 (19.3)	
Frequently	601 (6.5)	1868 (6.8)	
Smoking, %			<0.001
Never	7983 (87)	23 494 (85.4)	
Occasional	210 (2.3)	654 (2.4)	
Frequently	985 (10.7)	3358 (12.2)	
Cardiovascular disease, %			<0.001
Yes	617 (6.7)	1106 (4.0)	
No	8561 (93.3)	26 400 (96.0)	
Family history of diabetes, %			<0.001
Yes	1946 (21.2)	4482 (16.3)	
No	9232 (78.8)	23 024 (83.7)	

*Note*: Values were expressed as medians (quartile interval) or *n* (%).

Abbreviations: 2‐h PBG, 2‐h postprandial blood glucose; ALT, alanine transferase; AST, aspartate transferase; BMI, body mass index; DBP, diastolic blood pressure; eGFR, estimated glomerular filtration rate; FBG, fasting blood glucose; GGT, gamma‐glutamyl transferase; HbA1c, glycated hemoglobin; HDL‐C, high‐density lipoprotein cholesterol, HOMA‐IR, homeostasis model assessment of insulin resistance; LDL‐C, low‐density lipoprotein cholesterol; Non‐HDL‐C non‐high‐density lipoprotein‐cholesterol, RC, remnant cholesterol; SBP, systolic blood pressure; TC, total cholesterol; TG, triglyceride.

Among the total population (Table S1), when HOMA‐IR was used as a continuous variable, only HDL‐C and eGFR were negatively correlated with HOMA‐IR, while RC and the rest of the lipid parameters, FBG, 2‐hPBG, HbA1c, ALT, AST, GGT, age, BMI, SBP, and DBP were positively correlated with HOMA‐IR. Among females (Table S3), the same conclusion was reached when age was used as a control variable. Among males (Table S2), when age was used as a control variable, LDL‐C was not correlated with HOMA‐IR, and the rest of the findings were unchanged.

### 3.2 Relationship of each lipid parameter with diabetes

With adjustment for potential confounders (Table 3), RC (OR 1.417, 95% CI: 1.345–1.492), TC (OR 1.031, 95% CI: 1.006–1.058), TG (OR 1.200, 95% CI: 1.175–1.226), non‐HDL‐C (OR 1.113, 95% CI: 1.083–1.144), TG/HDL‐C (OR 1.158, 95% CI: 1.137–1.179), TC/HDL‐C (OR 1.266, 95% CI: 1.232–1.300), and LDL‐C/HDL‐C (OR 1.260, 95% CI: 1.214–1.307) were positively related to diabetes, HDL‐C (OR 0.492, 95% CI: 0.450–0.538) was negatively related to diabetes, and LDL‐C (*p* = 0.355) and diabetes were not associated with each other.

**TABLE 3:** Association of lipid parameters and diabetes, prediabetes, and insulin resistance.

	Diabetes^a^				Prediabetes^b^				Insulin resistance^c^			
	Diabetes/total population 9416/36 684				Prediabetes/No diabetes subjects 20 442/27 268				Insulin resistance/total population 9174/36 684			
	Unadjusted		Adjusted		Unadjusted		Adjusted		Unadjusted		Adjusted	
	OR	*p* value	OR	*p* value	OR	*p* value	OR	*p* value	OR	*p* value	OR	*p* value
	(95% CI)		(95% CI)		(95% CI)		(95% CI)		(95% CI)		(95% CI)	
TC	1.049	<0.001	1.031	0.02	1.208	<0.001	1.235	<0.001	1.127	<0.001	0.985	0.317
	(1.027, 1.071)		(1.006, 1.058)		(1.177, 1.240)		(1.198, 1.274)		(1.104, 1.151)		(0.957, 1.014)	
TG	1.346	<0.001	1.2	<0.001	1.493	<0.001	1.274	<0.001	1.602	<0.001	1.292	<0.001
	(1.319, 1.373)		(1.175, 1.226)		(1.436, 1.553)		(1.226, 1.324)		(1.566, 1.639)		(1.260, 1.324)	
HDL‐C	0.37	<0.001	0.492	<0.001	0.63	<0.001	0.801	<0.001	0.254	<0.001	0.271	<0.001
	(0.343, 0.399)		(0.450, 0.538)		(0.581, 0.684)		(0.729, 0.880)		(0.235, 0.275)		(0.243, 0.302)	
LDL‐C	1.034	0.01	1.015	0.36	1.255	<0.001	1.267	<0.001	1.138	<0.001	0.984	0.385
	(1.007, 1.061)		(0.984, 1.047)		(1.215, 1.297)		(1.220, 1.316)		(1.109, 1.169)		(0.950, 1.020)	
Non‐HDL‐C	1.172	<0.001	1.113	<0.001	1.35	<0.001	1.329	<0.001	1.323	<0.001	1.107	<0.001
	(1.145, 1.199)		(1.083, 1.144)		(1.311, 1.391)		(1.284, 1.375)		(1.293, 1.354)		(1.073, 1.142)	
RC	1.783	<0.001	1.417	<0.001	1.99	<0.001	1.555	<0.001	2.191	<0.001	1.488	<0.001
	(1.700, 1.870)		(1.345, 1.492)		(1.843, 2.149)		(1.438, 1.682)		(2.086, 2.302)		(1.404, 1.577)	
TG/HDL‐C	1.281	<0.001	1.158	<0.001	1.399	<0.001	1.204	<0.001	1.494	<0.001	1.243	<0.001
	(1.258, 1.305)		(1.137, 1.179)		(1.348, 1.451)		(1.163, 1.247)		(1.463, 1.525)		(1.216, 1.271)	
TC/HDL‐C	1.446	<0.001	1.266	<0.001	1.517	<0.001	1.358	<0.001	1.75	<0.001	1.41	<0.001
	(1.412, 1.481)		(1.232, 1.300)		(1.467, 1.568)		(1.310, 1.408)		(1.706, 1.795)		(1.367, 1.455)	
LDL‐C/HDL‐C	1.444	<0.001	1.26	<0.001	1.573	<0.001	1.419	<0.001	1.806	<0.001	1.431	<0.001
	(1.398, 1.492)		(1.214, 1.307)		(1.508, 1.641)		(1.356, 1.486)		(1.747, 1.868)		(1.372, 1.494)	

Abbreviations: 2‐h PBG, 2‐h postprandial blood glucose; ALT, alanine transferase; AST, aspartate transferase; BMI, body mass index; CI, confidence interval; CVD, cardiovascular diseases; DBP, diastolic blood pressure; eGFR, estimated glomerular filtration rate; FBG, fasting blood glucose; GGT, gamma‐glutamyl transferase; HbA1c, glycated hemoglobin; HDL‐C, high‐density lipoprotein cholesterol; LDL‐C, low‐density lipoprotein cholesterol; OR odds ratio; RC, remnant cholesterol; SBP, systolic blood pressure; TC, total cholesterol; TG, triglyceride.

^a^ Adjusted for age, sex, center, BMI, ALT, AST, GGT, SBP, DBP, eGFR, smoking habits, family history of diabetes, and cardiovascular disease.

^b^ Adjusted for age, sex, center, BMI, ALT, AST, GGT, SBP, DBP, eGFR, family history of diabetes, and cardiovascular disease.

^c^ Adjusted for age, sex, center, BMI, ALT, AST, GGT, SBP, DBP, eGFR, smoking habits, family history of diabetes, cardiovascular disease, FBG, 2‐h PBG, and HbA1c.

### 3.3 Relationship of each lipid parameter with prediabetes

After potential confounders were adjusted (Table 3), RC (OR 1.555, 95% CI: 1.438–1.682), TC (OR 1.235, 95% CI: 1.198–1.274), TG (OR 1.274, 95% CI: 1.226–1.324), LDL‐C (OR 1.267, 95% CI: 1.220–1.316), non‐HDL‐C (OR 1.329, 95% CI: 1.284–1.375), TG/HDL‐C (OR 1.204, 95% CI: 1.163–1.247), TC/HDL‐C (OR 1.358, 95% CI: 1.310–1.408), and LDL‐C/HDL‐C (OR 1.419, 95% CI: 1.356–1.486) were positively associated with prediabetes and HDL‐C (OR 0.801, 95% CI: 0.729–0.880) was negatively correlated with prediabetes.

### 3.4 Relationship of each lipid parameter with IR

After potential confounders were adjusted (Table 3), RC (OR 1.488, 95% CI: 1.404–1.577), TG (OR 1.292, 95% CI: 1.260–1.324), non‐HDL‐C (OR 1.107, 95% CI: 1.073–1.142), TG/HDL‐C (OR 1.243, 95% CI: 1.216–1.271), TC/HDL‐C (OR 1.410, 95% CI: 1.367–1.455), and LDL‐C/HDL‐C (OR 1.431, 95% CI: 1.372–1.494) were positively associated with IR and HDL‐C (OR 0.271, 95% CI: 0.243–0.302) had a negative relation with IR.

LDL‐C (*p* = 0.385) and TC (*p* = 0.317) revealed no relationship with IR.

### 3.5 Stratified analyses of RC and diabetes

From Table 4, it can be seen that RC had positive association with diabetes in both sexes (women: OR 1.523, 95% CI: 1.427–1.625; men: OR 1.203, 95% CI: 1.100–1.316) and at any level of BMI (BMI <24 kg/m^2^: OR 1.536, 95% CI: 1.404–1.682; BMI 24–28 kg/m^2^: OR 1.401, 95% CI: 1.300–1.510; and BMI ≥28 kg/m^2^: OR 1.272, 95% CI: 1.127–1.435), age (age <60 years old: OR 1.515, 95% CI: 1.415–1.622; age ≥60 years old: OR 1.263, 95% CI: 1.166–1.367), or blood pressure (with hypertension: OR 1.345, 95% CI: 1.252–1.445; without hypertension: OR 1.473, 95% CI: 1.367–1.589).

**TABLE 4:** Association between RC and diabetes and insulin resistance according to sex, age, blood pressure, and BMI.

	Diabetes^a^		Insulin resistance^b^	
Subgroup	Adjusted OR (95% CI)	*p* value	Adjusted OR (95% CI)	*p* value
Sex				
Women (*n* = 25 363)	1.523 (1.427, 1.625)	<0.001	1.448 (1.350, 1.554)	<0.001
Men (*n* = 11 321)	1.203 (1.100, 1.316)	<0.001	1.509 (1.360, 1.675)	<0.001
BMI (kg/m^2^)				
<24 (*n* = 17 145)	1.536 (1.404, 1.682)	<0.001	1.678 (1.505, 1.872)	<0.001
24–28 (*n* = 14 228)	1.401 (1.300, 1.510)	<0.001	1.419 (1.309, 1.538)	<0.001
≥28 (*n* = 5311)	1.272 (1.127, 1.435)	<0.001	1.230 (1.074, 1.408)	0.003
Age (years old)				
<60 (*n* = 22 896)	1.515 (1.415, 1.622)	<0.001	1.617 (1.499, 1.740)	<0.001
≥60 (*n* = 13 788)	1.263 (1.166, 1.367)	<0.001	1.325 (1.209, 1.452)	<0.001
Hypertension^c^				
Yes (*n* = 14 726)	1.345 (1.252, 1.445)	<0.001	1.412 (1.301, 1.531)	<0.001
No (*n* = 21 958)	1.473 (1.367, 1.589)	<0.001	1.553 (1.429, 1.687)	<0.001

Abbreviations: 2‐h PBG, 2‐h postprandial blood glucose; ALT, alanine transferase; AST, aspartate transferase; BMI, body mass index; CI, confidence interval; CVD, cardiovascular diseases; DBP, diastolic blood pressure; eGFR, estimated glomerular filtration rate; FBG, fasting blood glucose; GGT, gamma‐glutamyl transferase; HbA1c, glycated hemoglobin; HDL‐C, high‐density lipoprotein cholesterol, LDL‐C, low‐density lipoprotein cholesterol; OR, odds ratio; RC, remnant cholesterol; SBP, systolic blood pressure; TC, total cholesterol; TG, triglyceride.

^a^ Adjusted for age, sex, center, BMI, ALT, AST, GGT, SBP, DBP, eGFR, smoking habits, family history of diabetes, and cardiovascular disease.

^b^ Adjusted for age, sex, center, BMI, ALT, AST, GGT, SBP, DBP, eGFR, smoking habits, family history of diabetes, cardiovascular disease, FBG, 2‐h PBG, and HbA1c.

^c^ Hypertension was defined as SBP ≥140 mmHg or DBP ≥90 mmHg or self‐reported hypertension.

### 3.6 Stratified analyses of RC and prediabetes

In Table 5, we see that RC was positively related to prediabetes in both sexes (women: OR 1.565, 95% CI: 1.423–1.722; men: OR 1.477, 95% CI: 1.284–1.699) and at any level of BMI (BMI <24 kg/m^2^: OR 1.559, 95% CI: 1.393–1.745; BMI 24–28 kg/m^2^: OR 1.658, 95% CI: 1.464–1.877; BMI ≥28 kg/m^2^: OR 1.306, 95% CI: 1.032–1.652), age (age <60 years old: OR 1.708, 95% CI: 1.556–1.874; age ≥60 years old: OR 1.328, 95% CI: 1.147–1.539), or blood pressure (with hypertension: OR 1.476, 95% CI: 1.293–1.685; without hypertension: OR 1.619, 95% CI: 1.469–1.784).

**TABLE 5:** Association between RC and prediabetes according to sex, age, blood pressure, and BMI.

	Prediabetes^a^	
Subgroup	Adjusted OR (95% CI)	*p* value
Sex		
Women (*n* = 19 451)	1.565 (1.423, 1.722)	<0.001
Men (*n* = 7817)	1.477 (1.284, 1.699)	<0.001
BMI (kg/m^2^)		
<24 (*n* = 13 851)	1.559 (1.393, 1.745)	<0.001
24–28 (*n* = 10 088)	1.658 (1.464, 1.877)	<0.001
≥28 (*n* = 3329)	1.306 (1.032, 1.652)	<0.001
Age (years old)		
<60 (*n* = 18 345)	1.708 (1.556, 1.874)	<0.001
≥60 (*n* = 8923)	1.328 (1.147, 1.539)	<0.001
Hypertension^b^		
Yes (*n* = 9560)	1.476 (1.293, 1.685)	<0.001
No (*n* = 17 708)	1.619 (1.469, 1.784)	<0.001

Abbreviations: 2‐h PBG, 2‐h postprandial blood glucose; ALT, alanine transferase; AST, aspartate transferase; BMI, body mass index; CI confidence interval; CVD, cardiovascular diseases; DBP, diastolic blood pressure; eGFR, estimated glomerular filtration rate; FBG, fasting blood glucose; GGT, gamma‐glutamyl transferase; HbA1c, glycated hemoglobin; HDL‐C, high‐density lipoprotein cholesterol, LDL‐C, low‐density lipoprotein cholesterol; OR odds ratio; RC, remnant cholesterol; SBP, systolic blood pressure; TG, triglyceride; TC, total cholesterol.

^a^ Adjusted for age, sex, center, BMI, AlT, AST, GGT, SBP, DBP, eGFR, family history of diabetes, and cardiovascular disease.

^b^ Hypertension was defined as SBP ≥140 mmHg or DBP ≥90 mmHg or self‐reported hypertension.

### 3.7 Stratified analyses of RC and IR

In Table 4, RC can be seen to be positively linked to IR in both sexes (women: OR 1.448, 95% CI: 1.350–1.554; men: OR 1.509, 95% CI: 1.360–1.675) and at any level of BMI (BMI < 24 kg/m^2^: OR 1.678, 95% CI: 1.505–1.872; BMI 24–28 kg/m^2^: OR 1.419, 95% CI: 1.309–1.538; BMI ≥28 kg/m^2^: OR 1.230, 95% CI: 1.074–1.408), age (age <60 years old: OR 1.617, 95% CI: 1.499–1.740; age ≥60 years old: OR 1.325, 95% CI: 1.209–1.452), or blood pressure (with hypertension: OR 1.412, 95% CI: 1.301–1.531; without hypertension: OR 1.553, 95% CI: 1.429–1.687).

### 3.8 Association of RC and diabetes, prediabetes, and IR in subjects with HDL‐C ≥1.0 mmol/L, TG <2.3 mmol/L, or LDL‐C <2.6 mmol/L

From Table 6, it is clear that the relationship between RC and diabetes still remained when TG <2.3 mmol/L (OR 1.256, 95% CI: 1.135–1.390), LDL‐C <2.6 mmol/L (OR 1.306, 95% CI: 1.203–1.418), or HDL‐C ≥ 1.0 mmol/L (OR 1.456, 95% CI: 1.366–1.550). RC and IR was still statistically related when TG <2.3 mmol/L (OR 1.278, 95% CI: 1.140–1.433), LDL‐C <2.6 mmol/L (OR 1.552, 95% CI: 1.416–1.701), or HDL‐C ≥1.0 mmol/L (OR 1.490, 95% CI: 1.389–1.598). From Table 7, RC and prediabetes can be seen to be still statistically relevant when TG <2.3 mmol/L (OR 1.503, 95% CI: 1.342–1.684), LDL‐C <2.6 mmol/L (OR 1.597, 95% CI: 1.418–1.798), or HDL‐C ≥1.0 mmol/L (OR 1.553, 95% CI: 1.421–1.697).

**TABLE 6:** Association between RC and diabetes and insulin resistance when TG <2.3, LDL‐C <2.3, and HDL‐C ≥1 mmol/L.

	Diabetes^a^		Insulin resistance^b^	
	Adjusted OR (95% CI)	*p* value	Adjusted OR (95% CI)	*p* value
HDL‐C ≥1 mmol/L (*n* = 31 000)	1.456 (1.366, 1.550)	<0.001	1.490 (1.389, 1.598)	<0.001
TG <2.3 mmol/L (*n* = 30 320)	1.256 (1.135, 1.390)	<0.001	1.278 (1.140, 1.433)	<0.001
LDL‐C <2.6 mmol/L (*n* = 12 754)	1.306 (1.203, 1.418)	<0.001	1.552 (1.416, 1.701)	<0.001

Abbreviations: 2‐h PBG, 2‐h postprandial blood glucose; ALT, alanine transferase; AST, aspartate transferase; BMI, body mass index; CI, confidence interval; CVD, cardiovascular diseases; DBP, diastolic blood pressure; eGFR, estimated glomerular filtration rate; FBG, fasting blood glucose; GGT, gamma‐glutamyl transferase; HbA1c, glycated hemoglobin; HDL‐C, high‐density lipoprotein cholesterol, LDL‐C, low‐density lipoprotein cholesterol; OR odds ratio; RC, remnant cholesterol; SBP, systolic blood pressure; TC, total cholesterol; TG, triglyceride.

^a^ Adjusted for age, sex, center, BMI, AlT, AST, GGT, SBP, DBP, eGFR, smoking habits, family history of diabetes, and cardiovascular disease.

^b^ Adjusted for age, sex, center, BMI, AlT, AST, GGT, SBP, DBP, eGFR, smoking habits, family history of diabetes, cardiovascular disease, FBG, 2‐h PBG, and HbA1c.

**TABLE 7:** Association between RC and prediabetes when TG <2.3, LDL‐C <2.3, HDL‐C ≥1 mmol/L.

	Prediabetes^a^	
	Adjusted OR (95% CI)	*p* value
HDL‐C ≥1 mmol/L (*n* = 23 519)	1.553 (1.421, 1.697)	<0.001
TG <2.3 mmol/L (*n* = 23 435)	1.503 (1.342, 1.684)	<0.001
LDL‐C <2.6 mmol/L (*n* = 9506)	1.597 (1.418, 1.798)	<0.001

Abbreviations: 2‐h PBG, 2‐h postprandial blood glucose; ALT, alanine transferase; AST, aspartate transferase; BMI, body mass index; CI confidence interval; CVD, cardiovascular diseases; DBP, diastolic blood pressure; FBG, fasting blood glucose; GGT, gamma‐glutamyl transferase; HbA1c, glycated hemoglobin; HDL‐C, high‐density lipoprotein cholesterol, LDL‐C, low‐density lipoprotein cholesterol; OR, odds ratio; RC, remnant cholesterol; SBP, systolic blood pressure; TC, total cholesterol; TG, triglyceride.

^a^ Adjusted for sex, BMI, ALT, AST, GGT, SBP, DBP, eGFR, family history of diabetes, and cardiovascular disease.

### 3.9 Mediation analysis

**FIGURE 1:**
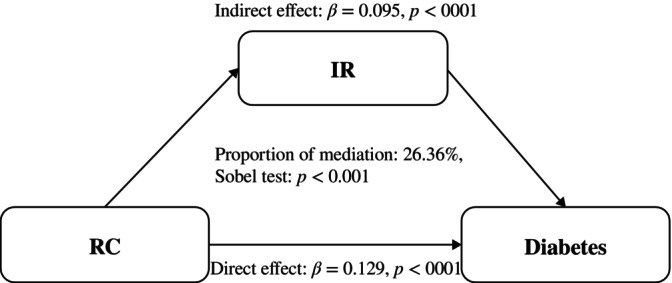
Mediation analysis of the relationship between RC and diabetes. IR, insulin resistance; RC, remnant cholesterol.

**FIGURE 2:**
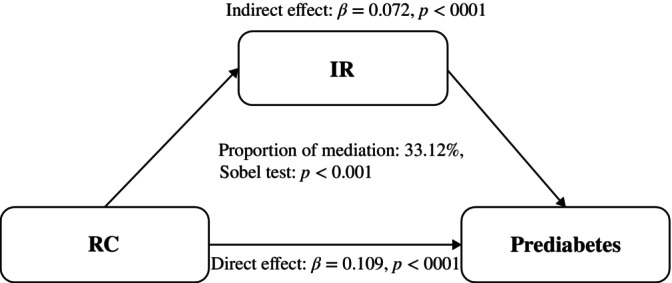
Mediation analysis of the relationship between RC and prediabetes. IR, insulin resistance; RC, remnant cholesterol.

Figure 1 depicts the indirect effect of IR between RC and diabetes with mediation is at 26.36%. Figure 2 depicts the indirect effect of IR between RC and prediabetes with mediation is at 33.12%.

### 3.10 Heterogeneity test

In Table S4, after adjusting for confounders, RC was associated with diabetes (OR 1.364, 95% CI: 1.266–1.469, *p* < 0.001), prediabetes (OR 1.604, 95% CI: 1.441–1.784, *p* < 0.001), and IR (OR 1.490, 95% CI: 1.372–1.618, *p* < 0.001) in the southern Chinese population and was similarly associated with diabetes (OR 1.468, 95% CI: 1.364–1.580, *p* < 0.001), prediabetes (OR 1.489, 95% CI: 1.326–1.673, *p* < 0.001), and IR (OR 1.483, 95% CI: 1.367–1.610, *p* < 0.001) in the northern Chinese population.

## 4 DISCUSSION

To our knowledge, this is the first large‐sample, multicenter study comparing the association between RC, conventional lipids, and lipid ratios with diabetes, prediabetes, and IR among the Chinese general population. Following major conclusions were drawn from the current study: (1) Novel lipid parameters generally have stronger positive correlations with diabetes, prediabetes, and IR than traditional lipids; (2) RC showed stronger positive correlations with diabetes, prediabetes, and IR than conventional lipids and lipid ratios; (3) HDL‐C was negatively related to diabetes, prediabetes, and IR; (4) RC was related to diabetes, prediabetes, and IR in the two genders and at any BMI, age and blood pressure levels, and in both southern and northern Chinese populations; (5) RC was still linked to diabetes, prediabetes, and IR even if TG, LDL‐C, or HDL‐C was within appropriate guideline advised levels; (6) significant indirect effect of IR between RC and diabetes and prediabetes with mediation.

The relationships of traditional lipid parameters and diabetes and prediabetes have been extensively investigated in the past.^24^, ^25^ TG and HDL‐C were repeatedly demonstrated as being connected with diabetes and prediabetes previously,^17^, ^26^, ^27^ but some controversies exist regarding the relationship of LDL‐C, TC and hyperglycemia.^24^ We have not found a link between LDL‐C and diabetes but only a weak correlation between TC and diabetes (OR = 1.031). Conclusions from different studies might differ due to ethnicity, sample size, and so on. The current study adds novel evidence to the relationship of conventional lipids and hyperglycemia. Unconventional lipid parameters, for instance, non‐HDL‐C, lipid ratios, and RC are popular issues lately.^24^ The relationships between lipid ratios and non‐HDL‐C and hyperglycemia were reviewed across numerous investigations,^17^, ^28^, ^29^, ^30^ whereas comparative researches on the correlation between these lipid parameters, especially RC and hyperglycemia are limited. The present study observed unconventional lipid parameters, especially RC, and had a stronger positive correlation with diabetes and prediabetes than traditional lipids. Unconventional lipid parameters offer additional risk details than traditional lipids and might better represent the interactions among lipid components.^31^

Very few investigations compared the relevance of RC and other lipid parameters with glycemic abnormalities. One study^24^ on a Japanese population of 15 464 subjects demonstrated a stronger positive relation between RC and diabetes risk than LDL‐C/HDL‐C, TC/HDL‐C, TG/HDL‐C, and TG, and they did not find any relevance of LDL‐C and TC and diabetes risk. However, in their study, 2‐h PBG was not detected, resulting in an underestimation of the prevalence of diabetes. Besides, generalizing results from other countries directly to China might not be appropriate. A research study of 11 557 coronary heart disease patients^32^ revealed that RC was more strongly positively correlated with diabetes and prediabetes than traditional lipid parameters and lipid ratios. Their study also did not test for 2‐h PBG, so their results were not accurate for determining glycemic status. In addition, their study only focused on a population with coronary heart disease, whereas the population of our survey was a huge sample of people from multiple regions of China, making the study population more representative. A report on 590 pregnant women^33^ concluded that it was RC, but not other traditional lipids, that was associated with gestational diabetes. Nevertheless, their study was only for specific types of diabetes and lipid ratios were not included. The current survey remedies the deficiencies of prior works and adds evidence to the strong relationship of RC and glycemic abnormalities. Moreover, we also conducted a stratified analysis, revealing that RC was closely correlated with diabetes and prediabetes irrespective of gender, blood pressure, age, and BMI status. This suggests that the relationship between RC and glycemic abnormalities is quite stable.

The rate at which RC leave the subendothelial space is very slow compared with the rate at which RC enter the subendothelial space, thus increasing the chances of macrophage infiltration and foam cell formation, which are more likely to contribute to atherosclerosis.^34^ Apolipoproteins on the surface of RC increase interactions with proteoglycans and are associated with lysophospholipids, partially digested TG, or minor amounts of lipids with cytotoxicity in the arterial wall and stimulate inflammation,^11^ leading to predisposition of RC to atherosclerosis. Arteriosclerosis causes the arterial blood vessel walls to thicken and harden, with a loss of elasticity and a narrowing of the lumen. This results in damages to the liver and pancreatic functioning,^35^ weakened hepatic glycogen synthesis,^36^, ^37^ reduced levels of insulin secretion,^38^ and elevated BG. On the other hand, RC is a critical component of cholesterol, accounting for one‐third of TC.^39^ Excessive islet cholesterol accumulation might account for elevated islet amyloid polypeptide aggregation and enhanced islet amyloid formation, which deteriorates pancreatic beta‐cell functionalities.^17^ Excess cholesterol also induces endoplasmic reticulum stress and mitochondrial function disorders, promotes reactive oxygen species of pancreatic beta‐cell production, eventually accounting for structural modification of insulin‐containing granules.^40^

Two previous small‐sample studies^41^, ^42^ demonstrated an independent correlation between RC and IR. Our study demonstrates firstly in a large Chinese general population that the positive correlation between RC and IR, which is known to be a major factor in causing hyperglycemia,^43^ is stronger than other traditional lipids and lipid ratios. This could also explain the reason for the stronger correlation of RC with diabetes and prediabetes than other lipid parameters. Additionally, we performed a mediator analysis and found that IR indeed mediated the relationship of RC and prediabetes and diabetes. Similarly, one study undertaken with 7308 Chinese by Hu et al.^40^ found IR to be a mediator in the relationship between RC and diabetes. Recently, it was indicated that abnormalities in the composition of TGRL might precede IR.^44^, ^45^, ^46^, ^47^ Some studies reported that the concentration and particle size of VLDL were strongly associated with IR in prediabetic patients.^44^, ^45^ Besides, IDL was demonstrated to be associated with cyclic kexin type 9 (PCSK9),^48^ which would mediate IR.^49^ On the other hand, IR also influences RC.^40^ IR of liver damages translocation of low‐density lipoprotein receptor‐associated protein 1 from intracellular vesicles to the plasma membrane of hepatocytes, causing a decrease in RC clearance,^50^ and thus creating a vicious cycle.

A recent cause‐and‐effect study has shown that higher circulating TG and LDL‐C concentrations considerably raised RC levels.^51^ Nevertheless, it is not clear whether RC is associated with hyperglycemia under normal control of traditional lipid levels. Therefore, we additionally analyzed the relationship of RC and diabetes, prediabetes, and IR while TG, HDL‐C, or LDL‐C were being retained in appropriate guideline advised levels, indicating that RC was still closely related to hyperglycemia and IR. Several clinical trials of statin therapy that target LDL‐C suggested that low LDL‐C concentrations were linked to increasing diabetes incidence.^52^ A Chinese population study^40^ found that it was the combination of low LDL‐C/high RC, instead of high LDL‐C/low RC, that was connected to diabetes, indicating that RC plays a role over LDL‐C. Indeed, per‐particle RC is more atherogenic than LDL‐C,^11^ which causes atherosclerosis rather than inflammation, while atherosclerosis's inflammatory element is motivated by RC.^37^, ^53^ Relationship between RC and inflammation might explain the residual hyperglycemia risk beyond LDL‐C.

Some Mendelian randomization analyses did not find evidence for a causality among TG and type 2 diabetes.^54^ Moreover, it has been described that high RC increases the risk of atherosclerosis also among people having normal TG values.^55^ TG is readily metabolized in most cells, while cholesterol is not easily broken down, so it is RC, not TG, that is the deleterious component of TGRL.^56^ Actually, TG might simply be a clinical surrogate for TGRL or RC, but they stand for different disorders of lipids, with RC being cholesterol in essence.^40^

It has been indicated that HDL‐C exerts anti‐hyperglycemic effects by protecting islet beta cells from glucose‐induced apoptosis.^52^ However, Mendelian randomization studies have described controversial findings about the relation of low HDL‐C and diabetes.^54^ A couple of clinical trials also found that HDL‐C elevation therapy failed to decrease the risk of contracting atherosclerotic disease significantly.^57^ In reality, RC accelerates HDL‐C reformulating into smaller, low‐cholesterol granules which would probably be short of atherosclerotic protection irrespective of HDL‐C levels.^58^ Therefore, the risk of hyperglycemia due to elevated RC should not be ignored in clinical practice, even if conventional lipid levels are kept within normal limits.

Firstly, this is the first investigation comparing the association between RC, conventional lipids, and lipid ratios with diabetes, prediabetes, and IR among a big sample of the Chinese people. Secondly, this is a multicenter study, so the study population could be representative of different regions in China. Thirdly, we measured FBG, 2‐h PBG (in particular, 2‐hPBG is difficult to obtain in large epidemiological studies), and HbA1c, which allowed us to diagnose diabetes and prediabetes more accurately than previous similar studies. However, this study still exhibits a few limitations. First, our study is cross‐sectional, so we are limited to drawing conclusions about correlation rather than causation. Secondly, RC values were calculated, not directly measured. Therefore, some bias might be present between the actual value and the calculated value.

## 5 CONCLUSIONS

In conclusion, we discovered that RC showed stronger positive associations with diabetes, prediabetes, and IR than conventional lipids and lipid ratios among the Chinese general population and was closely related to diabetes, prediabetes, and IR irrespective of gender, age, BMI, and blood pressure levels, and in both southern and northern Chinese populations. Additionally, we demonstrated that RC was closely related to diabetes, prediabetes, and IR even if LDL‐C, HDL‐C, or TG was within the appropriate guideline advised levels. RC could be calculated easily using the formula and is of clinical relevance, and we recommend that healthcare professionals consider RC as one of the major objectives of lipid management. Future clinical trials with RC as a core target of intervention need to be designed to explore if lowering RC reduces the risk of hyperglycemia.

## AUTHOR CONTRIBUTIONS

All authors read and approved final manuscript. Xin Zhou and Binqi Li conceived and designed this investigation. Binqi Li, Yang Liu, Lulu Chen, Li Yan, Xulei Tang, Zhengnan Gao, Qin Wan, Zuojie Luo, Guijun Qin and Guang Ning recruited participants and supervised the study. Binqi Li analyzed the data and wrote the paper's draft. Weijun Gu, Yiming Mu, Yang Liu, Xin Zhou, and Binqi Li devoted their time to the manuscript's writing, reviewing, and revising. Binqi Li, Yang Liu and Xin Zhou contributed equally to this work and share first authorship.

## FUNDING INFORMATION

The study is supported by Beijing Municipal Science and Technology Commission Project (Z201100005520014) and PLA General Hospital Youth Independent Innovation Science Fund project (22QNFC052).

## CONFLICT OF INTEREST STATEMENT

Lulu Chen and Guang Ning are Editorial Board members of Journal of Diabetes and co‐authors of the article. For the purpose of minimizing bias, they were excluded from all editorial decisions associated with acceptance of this article for publication.

## INFORMED CONSENT

Ethics approval and written informed consent were obtained from all participants before data collection.

## Supporting information


Table S1.

Table S2.

Table S3.

Table S4.

## Data Availability

For the protection of participants' privacy, these datasets are not freely available.
