# Supplementary material for: Remnant cholesterol is more positively related to diabetes, prediabetes, and insulin resistance than conventional lipid parameters and lipid ratios: A multicenter, large sample survey
Source: J Diabetes. 2024 Aug 13;16(8):e13592. doi: 10.1111/1753-0407.13592 (PMC11320755; doi:10.1111/1753-0407.13592)
Supplement: Supplementary file 1 — Table S1. [file JDB-16-e13592-s001.docx]

**Supplementary Table 1 Correlation matrix of continuous variables**

|  | HOMA-IR | HDL-C | LDL-C | TG | TC | Non-HDL-C | RC | TG/HDL-C | TC/HDL-C | LDL-C/HDL-C | FBG | 2hPBG | HbA1c | age | BMI | ALT | AST | GGT | eGFR | SBP | DBP |
| --- | --- | --- | --- | --- | --- | --- | --- | --- | --- | --- | --- | --- | --- | --- | --- | --- | --- | --- | --- | --- | --- |
| HOMA-IR | 1 |  |  |  |  |  |  |  |  |  |  |  |  |  |  |  |  |  |  |  |  |
| HDL-C | -0.243^**^ | 1 |  |  |  |  |  |  |  |  |  |  |  |  |  |  |  |  |  |  |  |
| LDL-C | 0.095^**^ | 0.346^**^ | 1 |  |  |  |  |  |  |  |  |  |  |  |  |  |  |  |  |  |  |
| TG | 0.394^**^ | -0.338^**^ | 0.145^**^ | 1 |  |  |  |  |  |  |  |  |  |  |  |  |  |  |  |  |  |
| TC | 0.098^**^ | 0.470^**^ | 0.888^**^ | 0.283^**^ | 1 |  |  |  |  |  |  |  |  |  |  |  |  |  |  |  |  |
| Non-HDL-C | 0.191^**^ | 0.203^**^ | 0.886^**^ | 0.437^**^ | 0.950^**^ | 1 |  |  |  |  |  |  |  |  |  |  |  |  |  |  |  |
| RC | 0.238^**^ | -0.172^**^ | 0.077^**^ | 0.711^**^ | 0.365^**^ | 0.465^**^ | 1 |  |  |  |  |  |  |  |  |  |  |  |  |  |  |
| TG/HDL-C | 0.412^**^ | -0.657^**^ | -0.022^*^ | 0.920^**^ | 0.037^**^ | 0.261^**^ | 0.626^**^ | 1 |  |  |  |  |  |  |  |  |  |  |  |  |  |
| TC/HDL-C | 0.346^**^ | -0.570^**^ | 0.445^**^ | 0.630^**^ | 0.397^**^ | 0.641^**^ | 0.526^**^ | 0.729^**^ | 1 |  |  |  |  |  |  |  |  |  |  |  |  |
| LDL-C/HDL-C | 0.276^**^ | -0.425^**^ | 0.653^**^ | 0.397^**^ | 0.456^**^ | 0.664^**^ | 0.208^**^ | 0.488^**^ | 0.899^**^ | 1 |  |  |  |  |  |  |  |  |  |  |  |
| FBG | 0.547^**^ | -0.156^**^ | 0.04^**^ | 0.201^**^ | 0.053^**^ | 0.107^**^ | 0.158^**^ | 0.224^**^ | 0.211^**^ | 0.160^**^ | 1 |  |  |  |  |  |  |  |  |  |  |
| 2hPBG | 0.411^**^ | -0.177^**^ | 0.015^**^ | 0.284^**^ | 0.035^**^ | 0.097^**^ | 0.195^**^ | 0.298^**^ | 0.219^**^ | 0.151^**^ | 0.576^**^ | 1 |  |  |  |  |  |  |  |  |  |
| HbA1c | 0.344^**^ | -0.129^**^ | 0.093^**^ | 0.238^**^ | 0.103^**^ | 0.157^**^ | 0.170^**^ | 0.243^**^ | 0.231^**^ | 0.190^**^ | 0.514^**^ | 0.527^**^ | 1 |  |  |  |  |  |  |  |  |
| age | 0.086^**^ | -0.037^**^ | 0.103^**^ | 0.106^**^ | 0.103^**^ | 0.128^**^ | 0.109^**^ | 0.099^**^ | 0.132^**^ | 0.120^**^ | 0.181^**^ | 0.234^**^ | 0.260^**^ | 1 |  |  |  |  |  |  |  |
| BMI | 0.500^**^ | -0.228^**^ | 0.088^**^ | 0.279^**^ | 0.057^**^ | 0.144^**^ | 0.149^**^ | 0.314^**^ | 0.298^**^ | 0.262^**^ | 0.239^**^ | 0.223^**^ | 0.190^**^ | 0.078^**^ | 1 |  |  |  |  |  |  |
| ALT | 0.274^**^ | -0.006^**^ | 0.118^**^ | 0.264^**^ | 0.149^**^ | 0.172^**^ | 0.142^**^ | 0.208^**^ | 0.142^**^ | 0.110^**^ | 0.129^**^ | 0.134^**^ | 0.094^**^ | 0.237^**^ | 0.237^**^ | 1 |  |  |  |  |  |
| AST | 0.089^**^ | 0.160^**^ | 0.119^**^ | 0.170^**^ | 0.194^**^ | 0.168^**^ | 0.147^**^ | 0.068^**^ | 0.012^**^ | -0.018^*^ | 0.024^**^ | 0.056^**^ | 0.027^**^ | 0.086^**^ | 0.086^**^ | 0.702^**^ | 1 |  |  |  |  |
| GGT | 0.326^**^ | -0.068^**^ | 0.169^**^ | 0.360^**^ | 0.209^**^ | 0.259^**^ | 0.243^**^ | 0.311^**^ | 0.021^**^ | 0.214^**^ | 0.213^**^ | 0.220^**^ | 0.172^**^ | 0.258^**^ | 0.258^**^ | 0.506^**^ | 0.410^**^ | 1 |  |  |  |
| eGFR | -0.117^**^ | -0.141^**^ | -0.232^*^ | -0.189^*^ | -0.273^*^ | -0.266^*^ | -0.155^*^ | -0.087^*^ | -0.110^*^ | -0.105^*^ | -0.046^*^ | -0.07^**^ | -0.11^**^ | -0.039^*^ | -0.039^*^ | -0.075^*^ | -0.182^*^ | -0.061^*^ | 1 |  |  |
| SBP | 0.273^**^ | -0.043^**^ | 0.132^**^ | 0.174^**^ | 0.141^**^ | 0.172^**^ | 0.122^**^ | 0.157^**^ | 0.175^**^ | 0.155^**^ | 0.272^**^ | 0.236^**^ | 0.164^**^ | 0.311^**^ | 0.311^**^ | 0.130^**^ | 0.104^**^ | 0.186^**^ | -0.125^*^ | 1 |  |
| DBP | 0.239^**^ | -0.095^**^ | 0.066^**^ | 0.188^**^ | 0.070^**^ | 0.111^**^ | 0.109^**^ | 0.188^**^ | 0.165^**^ | 0.136^**^ | 0.173^**^ | 0.125^**^ | 0.06^**^ | 0.280^**^ | 0.280^**^ | 0.154^**^ | 0.089^**^ | 0.201^**^ | -0.011^*^ | 0.645^**^ | 1 |

Note: ** P<0.001; * P<0.05

Abbreviations: ALT, alanine transferase; AST, aspartate transferase; BMI, body mass index; DBP, diastolic blood pressure; eGFR, estimated glomerular filtration rate; FBG, fasting blood glucose; GGT, gamma-glutamyl transferase; HbA1c, glycated hemoglobin; HDL-C, high-density lipoprotein cholesterol, HOMA-IR, homeostasis model assessment of insulin resistance; LDL-C, low-density lipoprotein cholesterol; Non-HDL-C non-high-density lipoprotein-cholesterol, RC, remnant cholesterol; 2h-PBG, 2-hour postprandial blood glucose; SBP, systolic blood pressure; TG, triglyceride; TC, total cholesterol
