# Supplementary material for: Remnant cholesterol is more positively related to diabetes, prediabetes, and insulin resistance than conventional lipid parameters and lipid ratios: A multicenter, large sample survey
Source: J Diabetes. 2024 Aug 13;16(8):e13592. doi: 10.1111/1753-0407.13592 (PMC11320755; doi:10.1111/1753-0407.13592)
Supplement: Supplementary file 2 — Table S2. [file JDB-16-e13592-s004.docx]

**Supplementary Table 2 Correlation matrix of continuous variables in male (after age was adjusted)**

|  | HOMA-IR | HDL-C | LDL-C | TG | TC | Non-HDL-C | RC | TG/HDL-C | TC/HDL-C | LDL-C/HDL-C | FBG | 2hPBG | HbA1c | BMI | ALT | AST | GGT | eGFR | SBP | DBP |
| --- | --- | --- | --- | --- | --- | --- | --- | --- | --- | --- | --- | --- | --- | --- | --- | --- | --- | --- | --- | --- |
| HOMA-IR | 1 |  |  |  |  |  |  |  |  |  |  |  |  |  |  |  |  |  |  |  |
| HDL-C | -0.112^*^ | 1 |  |  |  |  |  |  |  |  |  |  |  |  |  |  |  |  |  |  |
| LDL-C | 0.015 | 0.317^**^ | 1 |  |  |  |  |  |  |  |  |  |  |  |  |  |  |  |  |  |
| TG | 0.119^**^ | -0.252^*^ | -0.011 | 1 |  |  |  |  |  |  |  |  |  |  |  |  |  |  |  |  |
| TC | 0.101^*^ | 0.456^**^ | 0.880^**^ | 0.289^**^ | 1 |  |  |  |  |  |  |  |  |  |  |  |  |  |  |  |
| Non-HDL-C | 0.061^**^ | 0.190^**^ | 0.871^**^ | 0.397^**^ | 0.960^**^ | 1 |  |  |  |  |  |  |  |  |  |  |  |  |  |  |
| RC | 0.096^**^ | -0.177^**^ | -0.004 | 0.830^**^ | 0.392^**^ | 0.488^**^ | 1 |  |  |  |  |  |  |  |  |  |  |  |  |  |
| TG/HDL-C | 0.115^**^ | -0.423^**^ | -0.124^*^ | 0.933^**^ | 0.117^**^ | 0.261^**^ | 0.751^**^ | 1 |  |  |  |  |  |  |  |  |  |  |  |  |
| TC/HDL-C | 0.125^**^ | -0.544^**^ | 0.420^**^ | 0.591^**^ | 0.443^**^ | 0.659^**^ | 0.596^**^ | 0.656^**^ | 1 |  |  |  |  |  |  |  |  |  |  |  |
| LDL-C/HDL-C | 0.091^**^ | -0.423^**^ | 0.684^**^ | 0.485^**^ | 0.485^**^ | 0.668^**^ | 0.144^**^ | 0 | 0.852^**^ | 1 |  |  |  |  |  |  |  |  |  |  |
| FBG | 0.333^**^ | -0.04^**^ | 0.021^*^ | 0.129^**^ | 0.053^**^ | 0.071^**^ | 0.107^**^ | 0.112^**^ | 0.046^**^ | 0.046^**^ | 1 |  |  |  |  |  |  |  |  |  |
| 2hPBG | 0.271^**^ | -0.071^**^ | 0.000 | 0.140^**^ | 0.032^*^ | 0.058^**^ | 0.118^**^ | 0.120^**^ | 0.051^**^ | 0.051^**^ | 0.727^**^ | 1 |  |  |  |  |  |  |  |  |
| HbA1c | 0.254^**^ | -0.071^**^ | 0.040^**^ | 0.126^**^ | 0.06^**^ | 0.088^**^ | 0.109^**^ | 0.110^**^ | 0.089^**^ | 0.089^**^ | 0.750^**^ | 0.724^**^ | 1 |  |  |  |  |  |  |  |
| BMI | 0.221^**^ | -0.224^**^ | 0.078^**^ | 0.185^**^ | 0.063^**^ | 0.140^**^ | 0.145^**^ | 0.180^**^ | 0.261^**^ | 0.231^**^ | 0.128^**^ | 0.126^**^ | 0.098^**^ | 1 |  |  |  |  |  |  |
| ALT | 0.131^**^ | -0.001 | 0.062^**^ | 0.196^**^ | 0.111^**^ | 0.122^**^ | 0.139^**^ | 0.147^**^ | 0.093^**^ | 0.049^**^ | 0.103^**^ | 0.155^**^ | 0.750^**^ | 0.185^**^ | 1 |  |  |  |  |  |
| AST | 0.041^**^ | 0.111^**^ | 0.019^*^ | 0.114^**^ | 0.089^**^ | 0.064^**^ | 0.096^**^ | 0.065^**^ | -0.025^*^ | -0.068^*^ | 0.019^*^ | 0.072^**^ | 0.001 | 0.049^**^ | 0.737^**^ | 1 |  |  |  |  |
| GGT | 0.070^**^ | 0.097^**^ | 0.043^**^ | 0.228^**^ | 0.148^**^ | 0.122^**^ | 0.194^**^ | 0.142^**^ | 0.047^**^ | -0.026^*^ | 0.107^**^ | 0.152^**^ | 0.072^**^ | 0.075^**^ | 0.394^**^ | 0.484^**^ | 1 |  |  |  |
| eGFR | -0.058^**^ | -0.114^**^ | -0.237^*^ | -0.155^*^ | -0.283^*^ | -0.276^*^ | -0.142^*^ | -0.087^*^ | -0.133^*^ | -0.129^*^ | -0.032^*^ | -0.022^*^ | -0.033^*^ | -0.047^*^ | -0.09^*^ | -0.072^*^ | -0.023^*^ | 1 |  |  |
| SBP | 0.113^**^ | 0.030^**^ | 0.099^**^ | 0099^**^ | 0.121^**^ | 0.124^**^ | 0.077^**^ | 0.059^**^ | 0.080^**^ | 0.060^**^ | 0.128^**^ | 0.113^**^ | 0.036^**^ | 0.284^**^ | 0.124^**^ | 0.067^**^ | 0.124^**^ | -0.047^*^ | 1 |  |
| DBP | 0.096^**^ | -0.012 | 0.065^**^ | 0.125^**^ | 0.09^**^ | 0.103^**^ | 0.095^**^ | 0.084^**^ | 0.261^**^ | 0.059^**^ | 0.095^**^ | 0.084^**^ | 0.021^*^ | 0.256^**^ | 0.126^**^ | 0.068^**^ | 0.126^**^ | -0.064^*^ | 0.710^**^ | 1 |

Note: ** P<0.001; * P<0.05

Abbreviations: ALT, alanine transferase; AST, aspartate transferase; BMI, body mass index; DBP, diastolic blood pressure; eGFR, estimated glomerular filtration rate; FBG, fasting blood glucose; GGT, gamma-glutamyl transferase; HbA1c, glycated hemoglobin; HDL-C, high-density lipoprotein cholesterol, HOMA-IR, homeostasis model assessment of insulin resistance; LDL-C, low-density lipoprotein cholesterol; Non-HDL-C non-high-density lipoprotein-cholesterol, RC, remnant cholesterol; 2h-PBG, 2-hour postprandial blood glucose; SBP, systolic blood pressure; TG, triglyceride; TC, total cholesterol
