# Supplementary material for: Remnant cholesterol is more positively related to diabetes, prediabetes, and insulin resistance than conventional lipid parameters and lipid ratios: A multicenter, large sample survey
Source: J Diabetes. 2024 Aug 13;16(8):e13592. doi: 10.1111/1753-0407.13592 (PMC11320755; doi:10.1111/1753-0407.13592)
Supplement: Supplementary file 3 — Table S3. [file JDB-16-e13592-s002.docx]

**Supplementary Table 3 Correlation matrix of continuous variables in female (after age was adjusted)**

|  | HOMA-IR | HDL-C | LDL-C | TG | TC | Non-HDL-C | RC | TG/HDL-C | TC/HDL-C | LDL-C/HDL-C | FBG | 2hPBG | HbA1c | BMI | ALT | AST | GGT | eGFR | SBP | DBP |
| --- | --- | --- | --- | --- | --- | --- | --- | --- | --- | --- | --- | --- | --- | --- | --- | --- | --- | --- | --- | --- |
| HOMA-IR | 1 |  |  |  |  |  |  |  |  |  |  |  |  |  |  |  |  |  |  |  |
| HDL-C | -0.151^*^ | 1 |  |  |  |  |  |  |  |  |  |  |  |  |  |  |  |  |  |  |
| LDL-C | 0.031^**^ | 0.304^**^ | 1 |  |  |  |  |  |  |  |  |  |  |  |  |  |  |  |  |  |
| TG | 0.197^**^ | -0.326^*^ | 0.009 | 1 |  |  |  |  |  |  |  |  |  |  |  |  |  |  |  |  |
| TC | 0.031^**^ | 0.456^**^ | 0.894^**^ | 0.239^**^ | 1 |  |  |  |  |  |  |  |  |  |  |  |  |  |  |  |
| Non-HDL-C | 0.084^**^ | 0.175^**^ | 0.889^**^ | 0.373^**^ | 0.956^**^ | 1 |  |  |  |  |  |  |  |  |  |  |  |  |  |  |
| RC | 0.125^**^ | -0.206^*^ | 0.010 | 0.795^**^ | 0.361^**^ | 0.467^**^ | 1 |  |  |  |  |  |  |  |  |  |  |  |  |  |
| TG/HDL-C | 0.192^**^ | -0.486^*^ | -0.119^*^ | 0.944^**^ | 0.060^**^ | 0.227^**^ | 0.724^**^ | 1 |  |  |  |  |  |  |  |  |  |  |  |  |
| TC/HDL-C | 0.187^**^ | -0.554^*^ | 0.445^**^ | 0.613^**^ | 0.445^**^ | 0.675^**^ | 0.596^**^ | 0.645^**^ | 1 |  |  |  |  |  |  |  |  |  |  |  |
| LDL-C/HDL-C | 0.146^**^ | -0.421^*^ | 0.702^**^ | 0.254^**^ | 0.506^**^ | 0.699^**^ | 0.172^**^ | 0.269^**^ | 0.870^**^ | 1 |  |  |  |  |  |  |  |  |  |  |
| FBG | 0.420^**^ | -0.115^*^ | 0.027^**^ | 0.176^**^ | 0.045^**^ | 0.087^**^ | 0.138^**^ | 0.169^**^ | 0.164^**^ | 0.115^**^ | 1 |  |  |  |  |  |  |  |  |  |
| 2hPBG | 0.382^**^ | -0.158^*^ | 0.004 | 0.220^**^ | 0.024^**^ | 0.079^**^ | 0.164^**^ | 0.208^**^ | 0.188^**^ | 0.126^**^ | 0.746^**^ | 1 |  |  |  |  |  |  |  |  |
| HbA1c | 0.364^**^ | -0.112^*^ | 0.055^**^ | 0.183^**^ | 0.067^**^ | 0.110^**^ | 0.135^**^ | 0.167^**^ | 0.179^**^ | 0.140^**^ | 0.787^**^ | 0.710^**^ | 1 |  |  |  |  |  |  |  |
| BMI | 0.273^**^ | -0.205^*^ | 0.069^**^ | 0.165^**^ | 0.036^**^ | 0.107^**^ | 0.100^**^ | 0.164^**^ | 0.227^**^ | 0.212^**^ | 0.140^**^ | 0.164^**^ | 0.135^**^ | 1 |  |  |  |  |  |  |
| ALT | 0.153^**^ | 0 | 0.068^**^ | 0.146^**^ | 0.094^**^ | 0.104^**^ | 0.096^**^ | 0.112^**^ | 0.093^**^ | 0.071^**^ | 0.097^**^ | 0.132^**^ | 0.093^**^ | 0.143^**^ | 1 |  |  |  |  |  |
| AST | 0.073^**^ | 0.071^**^ | 0.041^**^ | 0.086^**^ | 0.085^**^ | 0.071^**^ | 0.075^**^ | 0.051^**^ | 0.038^**^ | -0.009 | 0.019^**^ | 0.058^**^ | 0.023^**^ | 0.058^**^ | 0.853^**^ | 1 |  |  |  |  |
| GGT | 0.131^**^ | 0.006 | 0.077^**^ | 0.165^**^ | 0.123^**^ | 0.135^**^ | 0.145^**^ | 0.112^**^ | 0.111^**^ | 0.074^**^ | 0.114^**^ | 0.140^**^ | 0.121^**^ | 0.087^**^ | 0.398^**^ | 0.373^**^ | 1 |  |  |  |
| eGFR | -0.059^*^ | -0.190^*^ | -0.221^*^ | -0.140^*^ | -0.284^*^ | -0.251^*^ | -0.122^*^ | -0.051^*^ | -0.066^*^ | -0.067^*^ | -0.034^*^ | -0.011^*^ | -0.035^*^ | 0.001 | -0.073^*^ | -0.112^*^ | -0.066^*^ | 1 |  |  |
| SBP | 0.155^**^ | -0.033^*^ | 0.109^**^ | 0.116^**^ | 0.118^**^ | 0.141^**^ | 0.098^**^ | 0.092^**^ | 0.138^**^ | 0.126^**^ | 0.169^**^ | 0.170^**^ | 0.102^**^ | 0.259^**^ | 0.087^**^ | 0.031^**^ | 0.058^**^ | -0.011 | 1 |  |
| DBP | 0.125^**^ | 0.065^*^ | 0.086^**^ | 0.141^**^ | 0.096^**^ | 0.128^**^ | 0.113^**^ | 0.117^**^ | 0.149^**^ | 0.126^**^ | 0.100^**^ | 0.102^**^ | 0.054^**^ | 0.243^**^ | 0.086^**^ | 0.043^**^ | 0.065^**^ | -0.036^*^ | 0.683^**^ | 1 |

Note: ** P<0.001; * P<0.05

Abbreviations: ALT, alanine transferase; AST, aspartate transferase; BMI, body mass index; DBP, diastolic blood pressure; eGFR, estimated glomerular filtration rate; FBG, fasting blood glucose; GGT, gamma-glutamyl transferase; HbA1c, glycated hemoglobin; HDL-C, high-density lipoprotein cholesterol, HOMA-IR, homeostasis model assessment of insulin resistance; LDL-C, low-density lipoprotein cholesterol; Non-HDL-C non-high-density lipoprotein-cholesterol, RC, remnant cholesterol; 2h-PBG, 2-hour postprandial blood glucose; SBP, systolic blood pressure; TG, triglyceride; TC, total cholesterol
