# Supplementary material for: Remnant cholesterol is more positively related to diabetes, prediabetes, and insulin resistance than conventional lipid parameters and lipid ratios: A multicenter, large sample survey
Source: J Diabetes. 2024 Aug 13;16(8):e13592. doi: 10.1111/1753-0407.13592 (PMC11320755; doi:10.1111/1753-0407.13592)
Supplement: Supplementary file 4 — Table S4. [file JDB-16-e13592-s003.docx]

**Supplementary Table 4 Heterogeneity test of association between RC and diabetes, prediabetes, and insulin resistance**

|  | **Diabetes ^a^** | | **Insulin resistance ^c^** | |
| --- | --- | --- | --- | --- |
|  | **Adjusted OR (95% CI)** | **Pvalue** | **Adjusted OR (95% CI)** | **Pvalue** |
| **Southern China (n=17628)** | 1.364(1.266,1.469) | <0.001 | 1.490(1.372,1.618) | <0.001 |
| **Northern China (n=19056)** | 1.468(1.364,1.580) | <0.001 | 1.483(1.367,1.610) | <0.001 |
|  | **Prediabetes ^b^** | |  |  |
|  | **Adjusted OR (95% CI)** | **Pvalue** |  |  |
| **Southern China (n=13518)** | 1.604(1.441,1.784) | <0.001 |  |  |
| **Northern China (n=13750)** | 1.489(1.326,1.673) | <0.001 |  |  |

a : adjusted for age, sex, center,BMI, AlT, AST, GGT, SBP, DBP, eGFR, smoking habits, family history of diabetes, cardiovascular disease

b : adjusted for age, sex, center,BMI, AlT, AST, GGT, SBP, DBP,eGFR, family history of diabetes, cardiovascular disease

c : adjusted for age,sex, center, BMI, AlT, AST, GGT, SBP, DBP, eGFR, smoking habits, family history of diabetes, cardiovascular disease, FBG, 2h-PBG, HbA1c

Abbreviations: ALT, alanine transferase; AST, aspartate transferase; BMI, body mass index; CVD, cardiovascular diseases; DBP, diastolic blood pressure; FBG, fasting blood glucose; GGT, gamma-glutamyl transferase; HbA1c, glycated hemoglobin; HDL-C, high-density lipoprotein cholesterol, LDL-C, low-density lipoprotein cholesterol; RC, remnant cholesterol; 2h-PBG, 2-hour postprandial blood glucose; SBP, systolic blood pressure; TG, triglyceride; TC, total cholesterol, OR odds ratio; CI confidence interva
